# Origin and Evolution of GALA-LRR, a New Member of the CC-LRR Subfamily: From Plants to Bacteria?

**DOI:** 10.1371/journal.pone.0001694

**Published:** 2008-02-27

**Authors:** Andrey V. Kajava, Maria Anisimova, Nemo Peeters

**Affiliations:** 1 Centre de Recherches de Biochimie Macromoléculaire, CNRS, University of Montpellier 1 and 2, Montpellier, France; 2 Institute of Computational Science, ETH Zurich, Zurich, Switzerland; 3 Swiss Institute of Bioinformatics, Lausanne, Switzerland; 4 Laboratoire des Interactions Plantes Micro-organismes (LIPM), UMR 441-2594 (INRA-CNRS), Castanet-Tolosan, France; University of Cape Town, South Africa

## Abstract

The phytopathogenic bacterium *Ralstonia solanacearum* encodes type III effectors, called GALA proteins, which contain F-box and LRR domains. The GALA LRRs do not perfectly fit any of the previously described LRR subfamilies. By applying protein sequence analysis and structural prediction, we clarify this ambiguous case of LRR classification and assign GALA-LRRs to CC-LRR subfamily. We demonstrate that side-by-side packing of LRRs in the 3D structures may control the limits of repeat variability within the LRR subfamilies during evolution. The LRR packing can be used as a criterion, complementing the repeat sequences, to classify newly identified LRR domains. Our phylogenetic analysis of F-box domains proposes the lateral gene transfer of bacterial GALA proteins from host plants. We also present an evolutionary scenario which can explain the transformation of the original plant LRRs into slightly different bacterial LRRs. The examination of the selective evolutionary pressure acting on GALA proteins suggests that the convex side of their horse-shoe shaped LRR domains is more prone to positive selection than the concave side, and we therefore hypothesize that the convex surface might be the site of protein binding relevant to the adaptor function of the F-box GALA proteins. This conclusion provides a strong background for further functional studies aimed at determining the role of these type III effectors in the virulence of *R. solanacearum.*

## Introduction

Leucine-rich repeats (LRRs) are 20–29-residue sequence motifs present in a number of proteins with diverse functions [1], [2]. In the 3D structures, each LRR corresponds to one coil of the solenoidal fold. The coils consist of a β-strand and mostly α-helical elements (can also be 3_10_ helix or polyproline helix) connected by loops. The coils are arranged so that all the strands and helices are parallel to a common axis, resulting in a non-globular, horseshoe-shaped molecule with a curved parallel β-sheet lining the inner circumference of the horseshoe and the helices flanking the outer circumference. In LRR proteins, a six-residue motif LxxLxL is conserved (x can be any amino acid and L-positions can be occupied by Leu, Val, Ile, and Phe), and in the known structures corresponds to a turn and a consecutive β-strand; whereas the remaining parts of repeats may be very different. While the invariant motif of the β-region is a characteristic feature of the entire LRR superfamily, the consensus sequences of the variable part suggest several specific subfamilies. LRR proteins can be subdivided into at least seven subfamilies [1], [3]. The repeats from different subfamilies retain a similar solenoidal fold and non-globular horseshoe shape but differ by 3D structures of individual repeats. Based on sequence analysis, it was concluded that LRRs from different subfamilies never occur concomitantly within one LRR protein [3]. This observation is explained by mutually exclusive inter-coil packing arrangement of LRRs from different subfamilies [3]. Such a relationship for LRRs suggests that LRR proteins of different subfamilies most probably have emerged independently during evolution rather than descended from a common ancestor. In line with this conclusion, the described LRR subfamilies could be assigned to a specific subgroup of eukaryotes or prokaryotes, and share similar functions and cellular locations [3]. For example, the bacterial LRR subfamily with the shortest known LRRs contains only extracellular proteins of Gram-negative bacteria. The Plant-Specific LRR (PS-LRR) subfamily has exclusively extracellular proteins from plants. Proteins of ribonuclease inhibitor-like LRR (RI-LRR) subfamily are intracellular and all belong to the Metazoa kingdom.

Since 1998, when these conclusions were formulated, a large number of new LRR proteins have been identified and several new 3D structures of LRR proteins have been determined [1]. After a lapse of nine years, the classification of the LRRs and most of the previously made conclusions, including the mutual exclusive rule, withstand the test of time. At the same time, the analysis of some newly identified LRRs shows that their assignment within the existing classification of the LRR subfamilies may lead to confusion.

Recently, it was shown that the phytopathogenic bacterium *Ralstonia solanacearum* encodes several type III effectors, called GALA proteins, that contain F-box and LRR domains [4], [5]. The F-box domain enables the interaction with SKP1 in the SCF-type E3 ubiquitin ligase protein complex [6]. Their LRRs (hereafter GALA-LRR) have a specific consensus pattern with characteristic differences from the previously described consensus sequences of LRR subfamilies, especially from the known bacterial LRR subfamilies. On the other hand, among the LRR subfamilies that are closest to *R. solanacearum* GALA-LRRs there is the Cysteine-Containing LRR (CC-LRR) subfamily of plant, animal and fungi proteins which can also contain the F-box domains and, therefore, may have a similar function. Thus, it was not clear, whether the GALA-LRR proteins are members of the CC-LRR subfamily or they should be assigned to a new LRR subfamily. Here we clarify this ambiguous case by using sequence analysis and molecular modeling. We also focus our analysis on the origin and evolution of GALA proteins from *R. solanacearum*.

## Results and Discussion

### Sequence analysis of GALA LRRs

**Figure 1:**
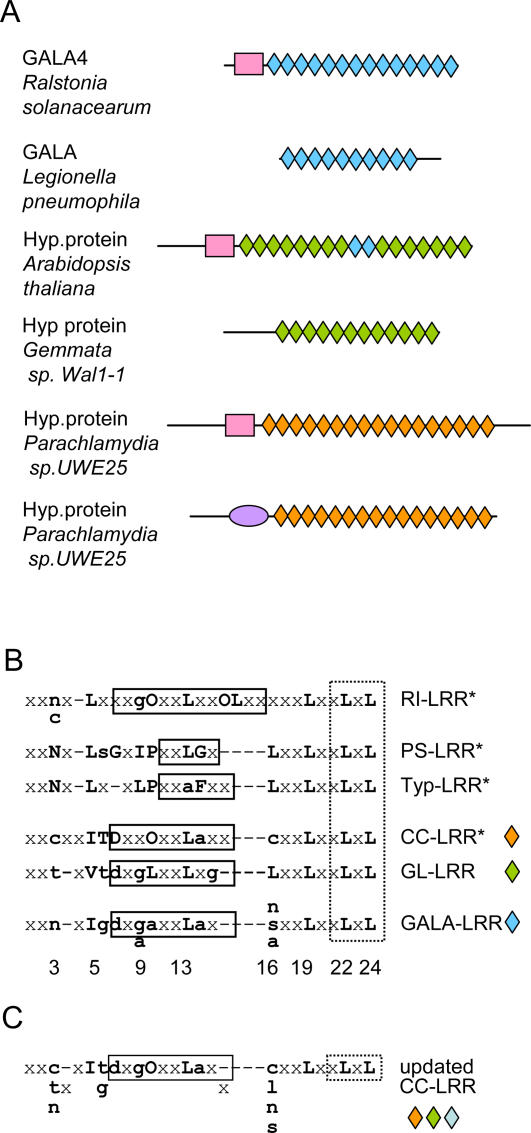
Specific consensus patterns of GALA-LRRs and GL-LRRs. (A) An arrangement of LRRs (rhombs), F-boxes (rectangles) and BTB domain (ellipse) within new representative proteins of CC-LRR subfamily. The following proteins are shown: GALA4 from *R. Solanacearum,* strain GMI1000 GenBank accession number CAD15502; GALA protein from *Legionella pneumophila* subsp.pneumophila str. Philadelphia 1, AAU27032; hypothetical protein from *Arabidopsis thaliana,* AAF82144; putative regulatory subunit from *Gemmata sp. Wa1-1*, AAX07517; hypothetical protein from *Parachlamydia sp. UWE25*, CAF23996; hypothetical protein from *Parachlamydia sp. UWE25,* CAF24006. (B) Alignment of some known (indicated by *) and newly identified LRR consensus sequences. (C) A consensus sequences of an updated CC-LRR. The boxes over the alignment outline known or putative α-helical and β-structural regions. Bold uppercase and lowercase letters indicate more than 60% and 20% identity, correspondingly. “O” denotes an apolar residue, “x” denotes any residue, “-“ is a position of a gap. Numbers below the GALA-LRR consensus sequence show the positions of conserved residues (see also Figure 2A).

Analysis of F-box containing GALA proteins from *Ralstonia solanacearum* shows that their 24-residue long LRRs have a specific consensus pattern that has characteristic differences (Figure 1) from the previously described LRRs [1], [3]. Comparison of GALA-LRRs with the other known 24-residue LRRs such as typical LRRs, PS-LRRs shows that GALA-LRRs frequently have Ile instead of Leu in position 5, Gly or Ala instead of Leu in position 9, Ala instead of Pro in position 10, and do not have a conserved Leu in position 16. The GALA-LRR consensus motif also has some differences with the 26-residue CC-LRR motif. For example, positions 3 and 16 of the GALA-LRR motif do not have a conserved Cys and position 6 is frequently occupied by Gly instead of Thr.

Using the generalized profile technique [7], a sensitive method for sequence database searches, we found the GALA-LRR type of repeats in about 40 proteins including proteins of the cucurbit crops pathogenic β-proteobacterium *Acidovorax avenae subsp. Citrulli*, the human pathogen, and γ-proteobacterium *Legionella pneumophila* [8], as well as in the aquatic planctomycete bacterium *Gemmata sp.* Wal 1. These proteins, unlike *R. solanacearum's* GALA proteins, don't contain an F-box domain. Sometimes their entire sequence corresponds to the LRR domain (Figure 1A). Some proteins have LRRs that are similar to GALA-LRR (GALA-like or GL-LRR hereafter), however, their consensus sequence has several characteristic differences from GALA-LRRs such as Val instead of Ile in position 5, Leu instead of Ala in position 10, presence of conserved Leu in position 16 (Figure 1B). Remarkably, isolated examples of GALA-LRR are found in GL-LRR domains of two F-box containing proteins from plants (Figure 1A). Sequence database searches with generalized profiles revealed GL-LRRs in more than a hundred LRR proteins. Among them are plant proteins, and also proteins from bacteria *(Gemmata sp. Wa1-1*, *Parachlamydia sp.*, *Legionella pneumophila*, *Rhodopirellula baltica)*, protists (*Entamoeba histolytica*, *Leishmania*, *Trypanosoma, Dictyostelium*) and animals (*Danio, Tetraodon, Drosophila, Anopheles gambiae, Xenopus, Strongylo, and Homo sapiens*). Interestingly, some of the GL-LRR proteins from plant, animal and protista also contain F-box domains.

### Place of GALA-LRRs and GL-LRRs in the classification of LRR proteins

Although, the newly identified GALA-LRRs and GL-LRRs do not perfectly fit any of the previously described consensus sequences of seven LRR subfamilies [1], [3], they have some similarities in the consensus sequences with CC-LRRs (Figure 1B). In particular, a characteristic ITD-motif of CC-LRRs (positions 5 to 7) is aligned with similar Igd- and Vtd-motifs of GALA- and GL-LRRs respectively. Furthermore, conserved apolar residues in positions 5, 10, 13, 19, 22 and 24 of the 24-residue-long GALA- and GL-LRRs can be aligned to the 26-residue-long CC-LRRs by deleting a residue in each of the two connecting loop regions of the CC-LRRs (Figure 1B). These loop regions are known to be the most accommodative for such length differences. Interestingly, many of the CC-LRR proteins, similarly to GALA-LRR and GL-LRR proteins, contain F-box domains (Figure 1A). Hence they can share functional similarity in that they recruit proteins, *via* their LRRs, to the SCF-type E3-ubiquitin ligase complex [6].

On the assumption of the membership of GALA and GL-LRRs in the CC-LRR subfamily, the previously proposed CC-LRR consensus [3] requires modifications. The updated CC-LRR consensus sequence is shown on Figure 1C. In this motif, Cys is not the only residue that occurs in positions 3 and 16: the other frequently occurring residues are Thr and Asn in position 3 and Leu, Asn and Ser in position 16. The updated CC-LRR often has Gly in addition to Thr in position 6. Finally, position 9 is frequently occupied by Gly. Database searches with the updated CC-LRR generalized profiles were able to detect such domains in heterogeneous group of about thousand proteins. Among these newly defined CC-LRR proteins there are not only proteins of animal or plant origin, but also proteins from pathogenic or non-pathogenic microorganisms such as bacteria (*Parachlamydia sp.),* protista (*Dictyostelium)* and fungi (*Cryptococcus, Candida albicans, Candida glabrata, Neurospora*). The sequence profiles and search results can be viewed on a dedicated web-page http://bioinfo.montp.cnrs.fr (Tools>Profiles>Show Profiles). Interestingly, about 600 of them have an F-box domain. This strongly supports a similar role for these specific LRRs in binding the protein substrate that is then recruited by the SCF-type E3-ubiquitin ligase.

### Structural implications

**Figure 2:**
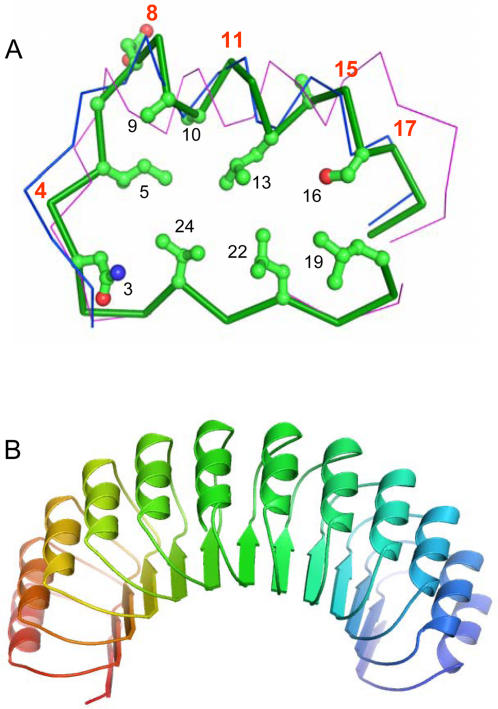
Structural model of GALA-LRR. (A) Cα-trace superposition of a modeled GALA-LRR and the known CC-LRR from human Skp2 protein [10] and RI-LRR from porcine ribonuclease inhibitor [46]. GALA-LRR model is shown in a ball-and-stick representation, CC-LRR is shown by a blue trace and RI-LRR by a magenta trace. Numbering of the conserved GALA-LRR residues is taken from Figure 1. Numbers in red point to positions inferred to be under positive selection. The carbon atoms are in green, oxygen in red, nitrogen in blue. (B) A ribbon diagram of a structural model of the C-terminal LRR domain of GALA4 type III effector protein from *R. solanacearum* (strain MolK2, region 170 to 460, accession code ZP_00946474). The figure was generated with Pymol [47]. The atomic coordinates of the model are available on request.

The knowledge of even one 3D protein structure in a given sequence alignment provides a powerful means to test the correctness of the whole alignment. In our case, the 3D structure of one CC-LRR protein, the human F-box protein Skp2 is known [9], [10] and was used to verify the alignment of GALA- and GL-LRRs with CC-LRRs. The analysis shows that all conserved and apolar residues of GALA- and GL-LRRs in the suggested alignment (positions 5, 10, 13, 19, 22 and 24) correspond to the residues of Skp2 CC-LRRs that form the hydrophobic core inside of the structure (Figures 1B and 2A). In the conserved position 3, GALA-LRRs have an Asn residue and GL-LRRs have a Thr residue instead of a Cys in CC-LRRs. This is an additional support for the alignment, because, in general, position 3 of LRRs tolerates a few amino acid residues including mentioned Asn, Thr and Cys. These residues being in position 3 can form specific hydrogen bonds with the peptide groups of the backbone. The two extra residues in the typical 26-residue-long CC-LRRs compared to the 24-residue-long GALA- and GL-LRRs, in the alignment are located in the loop regions of CC-LRRs connecting α-helices and β-strands (Figure 1B). The LRRs of Skp2 are variable in length and some of them are 1–2 residues shorter than typical 26-residue CC-LRR. The superposition of the 3D structures of these LRRs revealed that the loops are the most variable regions. In particular, missing residues of the short 24- and 25-residue LRRs of Skp2 are located in the loops. These structures represent good examples of how each of two loops of the CC-LRR can accommodate the loss of one residue. One of these short LRRs from Skp2 crystal structure (residue 2211 to 2235) was used as a template for construction of the GALA-LRR model (see Methods for details). Figure 2 shows structural models of a single GALA-LRR and a complete set of LRRs (12 repeats) from GALA4 of *Ralstonia solanacearum* (strain MolK2, personal communication C. Boucher and S. Genin). In Figure 2, the superposition of the GALA-LRR model and the crystal structure of Skp2 demonstrates that the difference between them is in the loops connecting α-helices and β-strands. The conserved asparagine of GALA-LRR (position 3 in Figure 2) similarly to the majority of the other LRR structures, located right after the β-strand so that it is able to form a network of specific hydrogen bonds with these NH and CO groups, thus satisfying their hydrogen-bonding potential in the hydrophobic core of the structure. The conserved bulky aliphatic residues form the hydrophobic core of the structure. The conserved small Ala and Gly residues allow a tighter side-by-side packing of α-helices.

**Figure 3:**
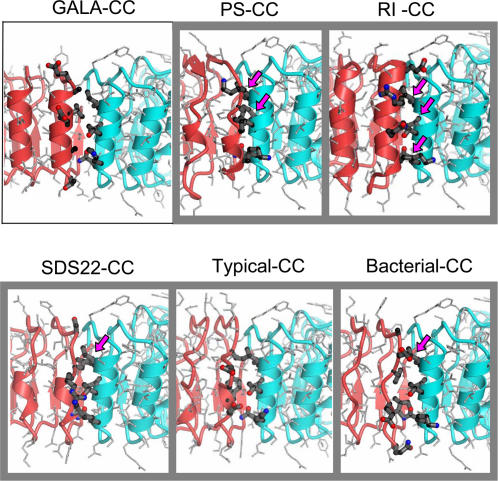
Side-by-side packing of LRRs having different consensus sequence motifs. “Knobs-into-holes” packing of GALA-LRRs against CC LRRs (contoured by a thin line box) and “knobs-into-knobs” packing of PS-LRRs, RI-LRRs, SDS22-LRRs, typical LRRs, bacterial LRRs against CC-LRRs. The CC-LRR-domain is in blue color. The side-chains that are involved in the inter-domain packing are shown by ball-and-stick representation. Arrows point at the clash sites where Cα and (or) Cβ atoms have distances between of 2.1–2.7 Å.

The modeling also shows that GALA-, GL-, and CC-LRRs can be packed well together and therefore, in contrast to the other LRR subfamilies, do not have mutually exclusive relationships with CC-LRRs in terms of inter-LRR packing (Figure 3). This conclusion is based on the following analysis. The conserved β-structural parts of the known crystal structures of LRR domains from different subfamilies and the model of GALA-LRRs were superimposed with the CC-LRR domain and the side-by-side packing of variable LRR fragments was analyzed (see Methods for details). The analysis shows that only the α-helices of GALA-LRR and CC-LRR have an energetically favorable “knobs-into-holes” interface while the superposition of LRRs from the other analyzed subfamilies with the CC-LRR results in “knobs-into-knobs” packing with steric tensions and voids (Figure 3). For example, the RI-LRR and CC-LRR, PS-LRR and CC-LRR, and SDS22-LRR and CC-LRR interfaces have distances between Cα and (or) Cβ atoms of 2.1-2.7 Å that are 0.5–1.1 Å closer than normally allowed limits for such distances [11]. This steric tension could be alleviated by a deformation of the LRR β-structure, but the distortion of the β-structural H-bonds would eventually also lead to the loss in the structure stability. The superposition of the typical LRR and CC-LRR domains does not lead to such close contacts, however, it results in an energetically unfavorable “knobs-into-knobs” packing with voids (Figure 3). Thus, our analysis suggests that some LRRs with different sequence motifs have an energetically favorable (“permissive”) packing, while simultaneous occurrence of the other ones in the same structure results in unfavorable (“mutually exclusive”) packing. The permissive packing of repeats with different consensus sequences may serve as a criterion for their membership in the same subfamily, at the same time as the mutually exclusive packing defines the boundaries between the LRR subfamilies.

It is worth mentioning that GALA-LRRs are erroneously assigned to the RI-LRR subfamily in the annotation of protein databases on the NCBI Web site (http://www.ncbi.nlm.nih.gov/entrez). In order to dissipate any doubt, Figure 2B displays the apparent difference of GALA-LRR and RI-LRR through the backbone superposition.

### Inferring origin of *R. solanacearum* GALA proteins

GALA F-box domains are functionally related to plant F-box domains [4]. Although some bacteria have a proteasome-like compartmentalized protease system [12], they do not have an ubiquitin-dependent protein degradation system like in eukaryotes. Still several bacteria have in their genome typical eukaryotic E3 ubiquitin ligase-like proteins among which F-box proteins, like the GALA proteins from *R. solanacearum* [13]. These bacterial F-box proteins also often contain eukaryote-like protein-protein interaction domains like LRR, ankyrin and WD40.

We systematically searched all the sequenced eubacterial genomes available (353 genomes available through TIGR Comprehensive Microbial Resource, release 24.0) for the presence of the F-box domain (automatic search with Pfam Hiden Markov Model for F-box (PF00646) service available at TIGR CMR). We only found F-box domains present in one chlamydiae species out of 11 complete sequence available (*Candidatus Protochlamydia amoebophila* strain UWE25) and in 9 proteobacteria out of 184 complete sequences available (alphaproteobacteria: *Mesorhizobium loti, Agrobacterium tumefaciens*; betaproteobacterium: *Ralstonia solanacearum*, gammaproteobacteria: *Pseudomonas syringae, Sodalis glossinidius, Coxiella burnetii, legionella pneumophila, Xanthomonas campestris* and *X. axonopodis*). All these positive hits correspond indeed to the presence of a canonical F-box domain. The evidence for functional F-box domains is available for both *A. tumefaciens* and *R. solanacearum* F-box containing proteins [13], [14]. A few low scoring hits (in proteins from *Borrelia burgdorferi, B. garinii, Chlamydophila caviae, Rhizobium etli, Salmonella tiphimurium* and *Streptococcus pneumoniae*) were inspected and clearly ruled out as being F-box domains (by constraints in primary sequence, see e.g. [15]).

Within the proteobacteria phylum, 9 out of 184 completely sequenced bacteria clearly contain at least one F-box-containing predicted protein. Among the 175 negatively scoring bacteria, we believe we can rule out the presence of “remnants” of F-box domain, which could have been indicative of gene loss. Considering such sporadic presence of this F-box domain, the scenario of systematic gene loss appears very unlikely

The F-box domain has its only described function in eukaryotic cells and is overrepresented in this kingdom (interpro F-box domain (IPR001810) hits: 735 in *A. thaliana,* 428 in *Caenorhabditis elegans,* 120 in humans, and only 46 hits among all bacteria sequence available, mostly in proteobacteria, see above). It is interesting to mention that all the bacteria containing F-box domains in their genome intimately interact with eukaryotes. For example, *P. amoebophila, S. glossinidius* and *M. loti* are symbionts of amoeba, insects and plants; *A. tumefaciens, R. Solanacearum, P. syringae, X. campestris* and *X. axonopodis* are plant pathogens and *C. burnetii* and *L. pneumophila* are human pathogens. Finally, for several of these F-box-containing bacterial proteins injection into their host cells (via specialised bacterial secretion systems) has been proven (*M. loti, A. tumefaciens, R. solanacearum*) [16]–[18] or predicted (*L. pneumophilae*) [8].

Among the seven *GALA* genes from the *R. solanacearum* genome (strain GMI1000, (http://bioinfo.genopole-toulouse.prd.fr/annotation/iANT/bacteria/ralsto/), *GALA1(*RSp0914) is located in an alternative codon usage region, *GALA2*(RSp0672) is flanked by a region duplicated elsewhere in the genome and *GALA3*(RSp0028) is flanked at either side by an alternative codon usage region. These genomic characteristic have been previously identified as potential signatures of LGT [19], [20]. Furthermore, considering the capacity of *R. solanacearum* to uptake DNA [21], it is natural to suggest a lateral gene transfer (LGT) from host plant DNA that gave rise to the F-box domain (and possibly the LRRs) of the GALA proteins.

One way of testing such a hypothesis is through phylogenetic analysis of the protein origins to identify putative donor for a potential LGT. Apart from the F-box domain, the F-box-containing proteins are constituted of repeat domains (mostly LRRs). Thus phylogenetic inference on the whole protein for large numbers of divergent taxa appears highly problematic. Instead we focused the analysis on the F-box domain, using 50 aa on their own, as well as together with 150 aa from the downsteam F-box-adjacent region or a shorter region containing 2 or 3 LRRs. Fifteen large datasets were assembled to include homologues from the broadest possible species range. The datasets varied by the similarity thresholds used in the profile searches [7], criteria used to align the sequences (profile, penalties), amount of gaps, and included from 217 to 2853 taxa (and from 64 to 217 aa in the alignment). Trees were inferred with neighbour-joining and maximum likelihood methods (see methods section). The resulted (approx.) 30 inferred phylogenies had low average branch supports, which demonstrated that phylogenetic inference for our data (with many short sequences of deep divergences and large percentage of gaps) is indeed problematic, even when considering the conserved F-box domain. The choice of the analysis model did not influence the inference much but considerably more variation in branching order was observed when analyzing different sets of sequences and alignments. In particular, varying the amount of gaps in the alignment had an impact. However, most phylogenies favoured the scenario where all *R. solanacearum* GALA genes clustered together and with *Arabidopsis thaliana* or with *Oryza sativa* as their closest basal lineages (for a representative example of an inferred Maximum Likelihood (ML) tree see Figure S1 that is a supplemental file phymlGALA.tre, which can be viewed with ATV-forester [22] from www.phylosoft.org/atv/). Only in some rare cases we also observed that one or two GALA genes were grouped with a non-plant lineage (but with low clade supports). Overall, the underlying phylogenetic signal appears to be in favour of the postulated LGT from a plant lineage. At the same time, the limited accuracy of inference does not enable us to confidently suggest a putative donor.

Our conclusion that the GALA-LRRs belong to the CC-LRR subfamily is consistent with the hypothesis of the lateral transfer. The updated CC-LRR subfamily has proteins from a very heterogeneous group of organisms including animals, plants, fungi, protista, and bacteria. By its wide taxonomic distribution it resembles the TpLRR subfamily [1] that includes proteins found in all three domains of life [23]. The broad distribution of TpLRR proteins also has been explained by LGT. Furthermore, the analysis of TpLRRs suggested that genes linked to pathogenicity can be shared between parasitic bacteria and parasitic eukaryotes [23]. The results of our present analysis of CC-LRRs agree with this hypothesis. The updated CC-LRR subfamily includes many proteins from bacteria (*Gemmata sp., Parachlamydia sp., Legionella pneumophila, Rhodopirellula baltica, Ralstonia solanacearum),* protista *(Entamoeba histolytica, Leishmania, Trypanosoma, Dictyostelium)* and fungi (*Cryptococcus, Candida albicans, Candida glabrata, Neurospora*), among which many are parasitic organisms colonizing plants and animals.

Despite the similarity of GALA-LRRs and CC-LRRs, they have some systematic differences (Figure 1B) and it needs to be explained. Usually, only about half of residue positions of LRRs remain conserved over a long evolutionary period [3]. The conservation usually reflects the importance of these residues for the preservation of the structure. However, GALA-LRRs are nearly perfectly repeated and this suggests that they emerged relatively recently. On the other hand, our molecular modeling indicates that GALA- and CC-LRRs fold in very similar structures that can be compatible and well-packed together, if a CC-LRR is inserted between GALA-LRR or *visa versa* (Figure 3). This conclusion is supported by the fact that two plant F-box-LRR proteins have a couple of GALA-LRRs inserted in GL-LRR tandem arrays (Figure 1A). Considering that CC-LRRs are much more abundant in plants than GALA-LRRs, and based on the above-mentioned facts, we propose the following sequence of evolutionary events that could “transform” the CC-LRR into GALA-LRR tandem arrays. First, the accumulation of point mutations may lead to the spontaneous occurrence of the first GALA-LRR and due to the structural complementarities between this new LRR and the CC-LRRs (see previous section) the occurrence of GALA-LRR does not significantly affect the overall structure and stability of the CC-LRR domain. Second, it is known that repetitive sequences can evolve more rapidly than non-repetitive ones [24], [25]. This applies both to the repeat multiplication and to the repeat deletion. Therefore, once appeared, GALA-LRRs can multiply and CC-LRRs disappear. As a result the plant CC-LRR genes, being acquired by a bacterium, may shed their CC-LRRs replacing them by GALA-LRRs seeded in their original sequences.

Currently it is not clear why *R. solanacearum* may prefer to generate arrays of GALA-LRRs instead of CC-LRRs. It has been shown that GALA are type III effectors required for virulence of *R. solanacearum* on three different plants, namely Arabidopsis, Tomato and *Medicago truncatula.* [13]. Furthermore it is very likely that the GALA type III effectors participate in virulence through their action in plant cells as the adaptors in SCF-type E3-ubiquitine ligases [4], [26]. In the SCF-type E3 ubiquitin ligase complex F-box containing proteins interact through their LRR (or other protein-protein interaction domains) with the protein targets to be ubiquitinated. We propose that a possible conversion from an original F-box and CC-LRR protein to an F-box and GALA-LRR protein was dictated by functional constrains. It is possible that the new GALA-LRRs have better plant-protein target recognition and are more versatile adaptors suitable to detect protein targets from diverse host plants.

### Testing GALA-LRRs for positive selection in an attempt to establish their functional binding sites

To gain insight into the function of the GALA proteins we examined whether adaptation could have acted on a proportion of protein residues during the evolution of GALA LRRs and identified positions of such sites, using likelihood ratio tests (LRTs) and the Bayesian prediction [27], [28]. In the agreement with the evolutionary scenario suggested by us for GALA-LRRs, data sets containing aligned LRRs were used to analyze the strength of selective pressure across its residues since their common ancestor sequence was acquired by the bacterium. To some extent, the evolution of LRRs in one particular GALA protein may be likened to the evolution of the gene family members after a duplication event whereby paralogous genes originate from the common single ancestral sequence [29]. Using this analogy we studied the process of the accumulation of substitutions at each site in a single LRR by comparing the codon substitutions at the homologous sites in other repeat sequences from the group of orthologous GALA proteins in four different *R. solanacearum* strains: GMI1000 [4], [20], RS1000 [30], UW551 [31] and MolK2 (C. Boucher and S. Genin, personal communication). The first two strains belong to the phylotype I and the others to the phylotype II, among the four phylotypes defined previously for this species complex [32].

The full coding DNA alignment of all LRRs from all the available GALA proteins (>400 sequences) was analyzed and none of the tests gave a significant evidence for positive selection, although parameter estimates hinted that this possibility could not be ruled out. This could mean that only for some GALAs the LRRs accumulated changes due to positive selection, as by averaging over all domains we loose power to detect positive selection affecting only certain GALA proteins. To test this we subdivided the aligned LRR sequences, so that only sequences from the orthologous GALAs were analyzed together. Parameter estimates from these alignments showed that for all GALA proteins 50–70% of the LRR positions are rather conserved while substitutions at remaining sites generally have neutral effect on the fitness of the protein. However for GALA2 both LRTs for positive selection were highly significant (with *P*-values <0.01). Estimates suggested that 8% of sites evolved under positive selection. For GALA2 LRRs, the Bayesian approach detected positions 8 and 15 (numbering as in Figures 1 and 2) with high probability (e.g., using model M8, the corresponding probabilities were 0.97 and 0.99, see Methods and Table S1 of Supporting Information for details). In accordance with the modeled GALA-LRR structure these residue positions are located in the α-helical region and exposed to the solution (Figure 2A). In the LRR domains these positions are located on the convex surface of the horseshoe shaped structure (Figure 2B).

For GALA7 LRRs, only the LRT comparing M7 vs. M8 supported positive selection (*P*-value <0.05), but the estimate of the ω ratio (describing selective pressure) was only slightly higher than 1 (ω≈1.15), indicating the lack of clear support for positive selection signal. Model M1a that does not allow positive selection described data equally as well as model M2a that allows positive selection. The Bayesian inference suggested that positions 4, 8, 11, 15 and 17 had a slightly elevated ratio of nonsynonymous to synonymous changes. Changes at these sites at the very least should be neutral to the fitness of the protein but may have a mild advantageous effect, possibly indicating a recent increase of adaptive pressure. The same can be concluded about position 4 in GALA1 and GALA3 LRRs and position 11 in GALA5 LRRs. If mapped on the structural model of the GALA-LRR, most of them (positions 8, 11, 15 and 17) are located on the external side of the α-helix and on the convex surface of the LRR solenoid. The side-chain in position 4 belongs to the loop connecting β-strand with the α-helix and also is exposed to the solvent (Figure 2A).

To see if signature of positive selection on GALA 2 and 7 is detectable on the level of the entire LRR domain of these proteins, we analyzed separately the groups of four GALA2 and GALA7 orthologous sequences from the different strains of *R. solanacearum*. Analysis of both GALA2 and GALA7 LRRs returned highly significant results for both tests (with *P*-values from 0.0014 to 0.025), providing the evidence of positive selection on both genes. The lack of the strong evidence for positive selection in GALA7 LRRs in the previous analysis suggests that positive selection may affect only certain (orthologous) repeats of GALA7 while the homologous sites in other repeats of this protein evolve neutrally. In this last analysis the number of sequences is too low for the Bayesian prediction to be accurate, and so the results of such inference are used only in an explorative manner, to see if the predicted positive selection sites correspond to any particular repeats and where such sites could be located. Mapping of the predicted sites of GALA2 onto the repeats of the LRR domain (with probability >0.85 from BEB) shows that they are located in position 15 in four LRRs and in position 21 of one LRR and dispersed over the LRR domain mostly on the convex surface (see Figures S2 and S3 of Supporting Information). Interestingly, the analysis of individual LRRs of GALA2 also pointed at position 15, that is the most represented in the analysis of the entire LRR domain. In the GALA7 LRR domain, these sites are also dispersed over the convex surface of the protein and are found in exactly the same positions as predicted in individual LRRs of GALA7 (positions 4, 11, 15 and 17).

Thus, it is encouraging to observe that most inferred positions throughout the LRR domain of orthologous GALAs coincide with those inferred in individual LRR repeats analysis on groups of GALA orthologues. It is important to mention that all positions, that are inferred to be under positive selection, are found on the surface of the structural model of GALA LRR domain. This grants additional support for the GALA-LRR structure prediction described above. Furthermore, our analysis suggests that the convex surface of the horse-shoe shaped GALA-LRR domain is more prone to positive selection than its concave one. It is tempting to propose that the selective pressure leading to an increase of variability on such residues could be the site of protein binding, relevant to the adaptor function of the F-box GALA proteins in the SCF-type E3 ubiquitin ligase. This study provides a strong background for further functional studies.

### Conclusions

The GALA-LRR example shows that the differences in LRR consensus motifs can be not only mutually exclusive in terms of inter-LRR packing as it was observed in LRRs from different subfamilies [3], but also permissive as we found in the case of GALA-LRRs and CC-LRRs. The permissive packing may serve as a criterion for the affiliation of LRRs having different consensus sequences with the same LRR subfamily. Therefore, one may expect to find a subfamily of evolutionary related proteins that share similar functions, cellular location, globular domains and, at the same time, having quite different repetitive consensus patterns. The relationships between GALA-, GL- and the other CC-LRRs suggest that structural constraints, namely, permissive packing of repeats may control the limits of the LRR variability within a subfamily. This result provides new insight into the fascinating interplay between the structural constraints and unusual evolutionary dynamics of LRRs and can be used to classify other newly identified LRR domains.

The *R. solanacearum* GALA proteins are bacterial F-box proteins containing a new kind of LRR, which can be found in other bacteria, plants and unicellular eukaryotes. These GALA-LRRs and related GL-LRRs are part of the CC-LRR subfamily, which is generally associated with an F-box domain. The presence of this F-box domain in GALA proteins is indicative of the probable ancient lateral transfer from eukaryotic (possibly plant) genes into a *R. solanacearum* ancestral recipient strain. We further looked into the GALA-LRR in all the GALA sequences available and found that for some GALA proteins there is a strong signature of positively selected residues and only on the convex side of the GALA-LRR structure. This suggests that the GALA proteins, probable E3-ubiquitin ligase adaptors, necessary for the virulence of *R. solanacearum* on its host plants, could bind their potential protein ligand on the convex side of the LRR domains, similarly to the *A. thaliana* TIR1 F-box type E3 ubiquitin ligase [33]. As we have an experimental system that enables us to test for the functionality of GALA proteins in virulence [4], this current study provides a strong theoretical background for testing the relevance of specific GALA-LRR residues to pathogenesis.

## Methods

### Sequence profile search

The generalized sequence profile method and the *pftools* package [7] were used. Since a single LRR would be unlikely to form a stable structure on its own, we limited the search to proteins containing at least three tandem repeats, thus increasing the selectivity of the search. Several profiles corresponding to different types of LRRs were constructed (GALA-LRR, GL-LRR and updated CC-LRR). The probability that a match is a product of chance alone was calculated by analyzing the score distribution obtained from a profile search against a regionally randomized version of the protein database, assuming an extreme value distribution [34]. All database searches were performed with a nonredundant data set constructed from 2006 releases of non-redundant protein sequence database including GenPept, Swissprot, PIR, PDF, PDB and NCBI RefSeq and available on the NCBI FTP site (ftp://ftp.ncbi.nih.gov/blast/db). The sequence profiles and results of the search can be viewed on a dedicated web-page http://bioinfo.montp.cnrs.fr/profiles (Tools/Profiles/Show Profiles).

### Molecular Modelling

The initial template for GALA-LRRs was taken from a 24-residue LRR (residues 2211 to 2235) of the known crystal structure of human Skp2 protein [9], [10] (see Results and Discussion) using the Insight II program [35]. The amino acid sequence of the template was edited in accordance with the GALA sequences using the homology modeling option of Insight II program. The structure was further refined by the energy minimization procedure based on the steepest descent algorithm implemented in the Discovery subroutine of Insight II, and tethering heavy backbone atoms to their starting conformations with force constant K = 100. The 300 steps of minimization led to a maximum RMS derivative of 0.4 kcal/(mol*Å). The next stage of minimization was 500 steps of the conjugate gradients algorithm, tethering the backbone atoms with lower force (K = 50), and then 300 steps with K = 25. The tethering was accompanied by setting the distance constraints at K = 50, in order to improve the geometry of H-bonds. To allay the concern that these constraints generated significant tensions in the minimized structure, the last calculation was performed without any restrictions, to an RMS derivative of 0.3 kcal/(mol*Å). The CVFF force field and the distance dependent dielectric constant were used for the energy calculations. The program PROCHECK [36] was used to check the quality of the modeled structure. In accordance with the PROCHECK results all residues of the LRR domain of the GALA4 model have backbone conformations from allowed regions of the Ramachandran plot; and G-factors of the polypeptide stereochemistry (a log-odds score based on the observed distribution of the covalent geometry) equal to −0.15. The overall average G-factors for the model is −0.49, values that would be expected for good-quality model.

To examine the side-by-side packing of LRRs from different subfamilies the following procedure was used. First, fragments of the LRR domains corresponding to different LRR subfamilies were extracted from the known crystal structures (CC-LRR, pdb code 2AST; Typical LRR, pdb code 2O6Q; Bacterial LRR, pdb code 1G9U; SDS22-like LRR, pdb code 1D0B; RI-LRR, pdb code 2BNH; PS-LRR, pdb code 1OGQ). Second, each of these structures and the structural model of GALA-LRRs were superimposed with CC-LRR domain. For the superposition, the conserved β-structural parts of the LRRs were used. Two adjacent β-strands of CC-LRR and the analyzed structures were superimposed and the side-by-side packing of the variable LRR fragments was analyzed.

### Tests for positive selection

The selective pressure at the protein level was measured by the ratio of nonsynonymous to synonymous rates *ω = d*_N_/*d*_S_, with *ω*<1, = 1, or >1 indicating conserved, neutral or adaptive evolution respectively [37]. Selective pressure was evaluated using the probabilistic Markov models of codon substitution [27], [28]. Such models describe the substitution process based on a multiple alignment tree. The transitions from one codon state to another are described by the transition probability matrix over time *t* as *P*(*t*) = exp(*Qt*). The generator matrix *Q* = {*q_ij_*} defines the instantaneous substitution rates at site *s* from codon *i* to codon *j* :
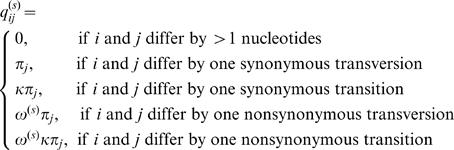



Here *π_j_* is the frequency of codon *j*, parameter *κ* is the transition/transversion ratio, and *ω* ^(*s*)^ is the *ω* ratio for site *s*. The codon substitution process is assumed independent among sites, and model parameters are estimated by maximizing the log-likelihood function of sequence data *X* = {*x*_s_} given a phylogeny with branch lengths τ and a model Μ:
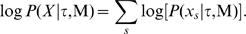



The models used in the analysis differed by statistical distributions of the *ω* ratio used to describe the variation of selective pressure along a sequence. Likelihood ratio test (LRT) for positive selection compares maximum log-likelihoods of two nested models, one of which allows sites under positive selection while another does not. To test that a model allowing positive selection describes data significantly better, twice the log-likelihood difference is compared to the χ^2^-distribution with degrees of freedom equal to the difference in the number of free parameters between the two models. We performed two LRTs for positive selection, comparing models M2a and M8 that allow sites with *ω*>1 (alternative hypotheses) with simpler models M1a and M7 respectively that do not allow sites with *ω*>1 (null hypotheses). Model M1a (nearly-neutral) assumes two site classes in proportions *p*_0_ and *p*_1_ = 1–*p*_0_: one with *ω*_0_ ratio estimated between 0 and 1, and the other with *ω*_1_ fixed at 1. The alternative model M2a (positive selection) extends the null model M1a by adding a proportion *p*_2_ of positively selected sites with *ω*_2_>1, estimated from data. The second LRT uses the null model M7 (beta) that assumes the *ω* ratio is drawn from a beta distribution defined between 0 and 1. The alternative model M8 has an extra class of sites under positive selection with *ω*>1.

We also considered two other codon models: the most simple one-ratio model M0, where *ω* is assumed to be constant over all sites in the sequence, and the discrete model M3 that allows three discrete classes of sites with ratios *ω*_0_, *ω*_1_, and *ω*_2_ occurring in proportions *p*_0_, *p*_1_ and *p*_2_ = 1−*p*_0_−*p*_1_. Models M0 and M3 are also nested, and can be used to perform the LRT for heterogeneity of selective pressure along the sequence [38]. This test is often significant, as most coding data has significantly heterogeneous selective pressures acting on different sites of the sequence, according to their functional importance and the role in the protein folding and stability. In comparison with models M8 and M2a, model M3 better combines the algorithmic simplicity with sufficient complexity necessary to reflect heterogeneity of selection pressure in nature. This model is often used to evaluate the underlying distribution of the selective pressure across sites in a sequence. Inconsistencies in estimates under different models may be a sign that the algorithm has not converged to a global optimum. To insure proper convergence, we performed repeated runs for each model (with different starting values) and confirmed that the distribution of selective pressure described by estimates under models M2a and M8 were compatible with the distribution estimated under M3 for all datasets analyzed.

Where a LRT for positive selective pressure was significant, we used the Bayesian inference to calculate posterior probabilities that a site belongs to a particular site class. The posterior distribution of the parameter of interest (in our case ω) is proportional to the product of its assumed prior distribution and the likelihood of the observed data given this prior. In this study we used the Bayesian Empirical Bayesian approach [28], where the posteriors are obtained by integrating over the prior distribution of selection-related parameters, while setting other model parameters to their maximum likelihood estimates. Sites with high posteriors probabilities (>0.95) of coming from a class with ω>1 are likely to have evolved under positive selection. Anisimova *et al*. [39] showed that the major factor affecting the accuracy Bayesian site prediction is the diversity of the data set and the number of sequences used. The sequence length was shown to have little effect on the accuracy of this method. We therefore believe that our LRRs analysis should have good accuracy as short sequence length may be compensated by the diversity and large numbers of sequences used in this study. Several site-by-site studies support this notion [40], [41]. While extreme levels of sequence divergence do not seem to compromise the accuracy of the LRTs, the Bayesian prediction becomes unreliable [28], [30]. In this study the divergence levels ranged from 0.21 to 0.34 nucleotide changes per codon per branch (calculated as the tree length divided by the number of branches in the unrooted tree; see Table S1). This corresponds to the optimal divergence levels and so insures good accuracy of Bayesian prediction results reported here.

All ML phylogenies were inferred using PHYML program [42]. The phylogenies used to perform the selection tests was inferred using coding sequences under HKY+Γ, and all phylogenies used for testing LGT hypothesis were inferred under WAG model [43]. Phylogenies for testing LGT hypothesis were also inferred using fast neighbor-joining method implemented in BIONJ [44], [45]. Branch supports were inferred using approximate LRT [45] and 100 bootstrap replicates where computation permitted.

## Supporting Information

Table S1 Maximum likelihood estimates of selection parameters for codon models
(0.06 MB DOC)

Figure S1 Phylogenetic tree supplemental file phymlGALA.tre, which can be viewed with ATV-forester from www.phylosoft.org/atv/
(0.04 MB TXT)

Figure S2 Positions of positive selection in GALA2
(0.03 MB DOC)

Figure S3 Positions of positive selection in GALA2
(0.02 MB DOC)
