# Supplementary material for: Origin and Evolution of GALA-LRR, a New Member of the CC-LRR Subfamily: From Plants to Bacteria?
Source: PLoS One. 2008 Feb 27;3(2):e1694. doi: 10.1371/journal.pone.0001694 (PMC2244805; doi:10.1371/journal.pone.0001694)
Supplement: Table S1 — Maximum likelihood estimates of selection parameters for codon models (0.06 MB DOC) [file pone.0001694.s001.doc]

**Suppl. Table S1: Maximum likelihood estimates of selection parameters for codon models M0, M1a, M2a, M3, M7 and M8**

| Domain | *T* | *S* | M0 | M3 | M1a | M2a | M7 | M8 |
| --- | --- | --- | --- | --- | --- | --- | --- | --- |
| Gala 1 | 31 | 15.15 | -1216.553559  κ=1.90, ω=0.37 | **-1175.218808***  κ = 2.27  p: 0.47 0.45 **0.09**  ω: 0.11 0.65 **1.76** | -1178.925429  κ = 2.20  p: 0.51 0.49  ω: 0.14 1.00 | -1177.81756  κ = 2.27  p: 0.50 0.45 **0.05**  ω: 0.15 1.00 **2.61** | -1179.108351  κ= 2.17  p= 0.85 q= 1.05 | -1177.510001  κ = 2.24  p_0_= 0.92  p= 0.99 q= 1.54  **p1= 0.08; ω= 1.74** |
| **Gala 2** | 108 | 58.15 | -3118.035287  κ = 2.94, ω= 0.30 | **-3025.346948***  κ = 3.35  p: 0.65 0.26 **0.08**  ω: 0.18 0.65 **1.73** | -3035.410085  κ = 3.32  p: 0.69 0.31  ω: 0.20 1.00 | **-3030.414669***  (*P*-value=0.007)  κ = 3.37  p: 0.67 0.24 **0.08**  ω: 0.21 1.00 **2.05** | -3025.194287  κ= 3.29  p= 1.08 q= 1.43 | **-3016.784310***  (*P*-value=1.2*10^-6^)  κ = 3.30  p_0_= 0.92  p= 1.43 q= 2.97  **p1= 0.08; ω=1.72** |
| Gala 3 | 54 | 28.26 | -1855.656152  κ =2.67, ω= 0.29 | **-1822.190469***  κ = 3.02  p: 0.63 0.33 **0.04**  ω: 0.18 0.61 1.62 | -1825.220628  κ= 3.03  p: 0.70 0.30  ω: 0.22 1.00 | -1825.220628  κ = 3.03  p: 0.70 0.20 **0.10**  ω: 0.22 1.00 **1.00** | -1824.319781  κ= 2.82  p= 1.46 q= 2.65 | -1822.869197  κ= 2.87  p_0_= 0.95  p= 1.78 q= 3.71  **p1= 0.05; ω= 1.43** |
| Gala 4 | 48 | 19.15 | -1362.421321  κ = 3.07, ω= 0.27 | **-1335.847149***  κ = 3.27  p: 0.44 0.52 **0.04**  ω: 0.10 0.45 **0.98** | -1345.917282  κ= 3.26  p: 0.51 0.49  ω: 0.17 1.00 | -1345.917282  κ = 3.26  p: 0.51 0.35 **0.14**  ω: 0.17 1.00 **1.00** | -1336.108627  κ= 3.24  p= 1.24 q= 3.25 | -1336.108627  κ = 3.25  p_0_= 1.00  p= 1.24 q= 2.62  **p_1_= 0.00; w= 1.00** |
| Gala 5 | 58 | 23.67 | -1656.503235  κ= 3.02, ω= 0.25 | **-1625.751200***  κ = 3.27  p: 0.50 0.45 0.04  ω: 0.12 0.41 1.43 | -1638.046057  κ = 3.21  p: 0.75 0.25  ω: 0.21 1.00 | -1638.046057  κ = 3.15  p: 0.69 0.29 0.02  ω: 0.21 1.00 1.82 | -1625.511206  κ = 3.19  p= 0.93 q= 2.25 | -1624.069927  κ = 3.23  p0= 0.96  p= 1.21 q= 3.61  p1= 0.04; ω= 1.42 |
| Gala 6 | 71 | 36.94 | -2670.543089  κ= 3.31, ω= 0.26 | **-2607.981903***  κ = 3.45  p: 0.50 0.50 0.00  ω: 0.11 0.46 17.61 | -2629.630276  κ= 3.35  p: 0.56 0.44  ω: 0.19 1.00 | -2629.201929  κ = 3.32  p: 0.55 0.42 0.03  ω: 0.20 1.00 1.73 | -2603.667117  κ= 3.49  p= 1.26 q= 2.78 | -2603.667125  κ = 3.49  p_0_= 1.00  p= 1.26 q= 2.78  p1= 0.00; ω= 2.00 |
| Gala 7 | 56 | 36.85 | -2100.761306  κ= 1.84, ω= 0.34 | **-2057.108275***  κ= 2.00  p: 0.52 0.48 0.00  ω: 0.18 0.71 13.42 | -2058.194569  κ = 2.07  p: 0.65 0.34  ω: 0.23 1.00 | -2058.194556  κ = 2.07  p: 0.65 0.21 0.13  ω: 0.23 1.00 1.00 | -2051.096915  κ = 2.05  p= 1.11 q= 1.45 | **-2047.545713***  **(P-value=0.029)**  κ = 2.10  p_0_= 0.79  p= 2.10 q= 5.38  **p1= 0.21 ω= 1.15** |

For each model the maximum log-likelihood values (the negative values) and the ML parameter estimates are presented. In red are the maximum log-likelihood values for models that fit data significantly better and positive selection estimates for those models. The significant P-values of the likelihood ratio tests are also in red and shown in brackets. The parameter estimates shown are transition/transversion ratio (κ) and parameters describing the ω-distribution (see methods section) with ω-values and corresponding proportions of sites.

*T* is the number of sequences used and *S* is the tree length measured by the number of expected substitutions per codon site along the whole phylogeny. Note that *S*/(2*T*-3) can be used as a measure of sequence divergence [38, 39].
