# Supplementary material for: Origin and Evolution of GALA-LRR, a New Member of the CC-LRR Subfamily: From Plants to Bacteria?
Source: PLoS One. 2008 Feb 27;3(2):e1694. doi: 10.1371/journal.pone.0001694 (PMC2244805; doi:10.1371/journal.pone.0001694)
Supplement: Figure S2 — Positions of positive selection in GALA2 (0.03 MB DOC) [file pone.0001694.s003.doc]

**Figure S2, Supplemental data, Kajava et al. 2008**

**Reference protein sequence and positions of sites under positive selection**

Highlighted sites were detected with all three models allowing positive selection (M2a, M3, and M8)

Yellow highlighting: a site was detected with a posterior probability > 0.95

Green highlighting: a site was detected with a posterior probability > 0.99

>UW551GALA2

111111111122222222223333333333444444444455555555556

123456789012345678901234567890123456789012345678901234567890

-------------------MVAPVSTHHAP---------------LPSAPAATDTGARHG

111111111111111111111

666666666777777777788888888889999999999000000000011111111112

123456789012345678901234567890123456789012345678901234567890

P-DQTGQQPAYAPARSSAGVALSPLGGLASLRLDAASAN----------TVCAP------

111111111111111111111111111111111111111111111111111111111111

222222222333333333344444444445555555555666666666677777777778

123456789012345678901234567890123456789012345678901234567890

-----RILPPAPCH-------------------DPRTAALQRVTHLSVHDRRALGELHHY

111111111111111111122222222222222222222222222222222222222222

888888888999999999900000000001111111111222222222233333333334

123456789012345678901234567890123456789012345678901234567890

PNLTSLQLEGNFTLQDLKALPATLRHLDLSACTG--GAKSFEAIAYLAGLP---------

222222222222222222222222222222222222222222222222222222222223

444444444555555555566666666667777777777888888888899999999990

123456789012345678901234567890123456789012345678901234567890

-LESLNVAGADIGDDGARLLAANPSLRALNAANGGIGAAGARALAESPVLASLDLTRNGI

333333333333333333333333333333333333333333333333333333333333

000000000111111111122222222223333333333444444444455555555556

123456789012345678901234567890123456789012345678901234567890

GDEGARALADSRSLTNLAVLNCLVTDVGARALAGNGTLTALDLGNLITETGNELEQAGYD

333333333333333333333333333333333333333444444444444444444444

666666666777777777788888888889999999999000000000011111111112

123456789012345678901234567890123456789012345678901234567890

RTANEITARGAWALAQNRSLTSLSIQGN-LCGDGGVQALAKNRTLTSLNVAYTDMTPASA

444444444444444444444444444444444444444444444444444444444444

222222222333333333344444444445555555555666666666677777777778

123456789012345678901234567890123456789012345678901234567890

TELARNPVLTSLSVRWNYGLGDAGVVELAKSPSLTLLDARSTGMGERATLALSANARIRV

444444444444444444455555555555555555555555555555555555555555

888888888999999999900000000001111111111222222222233333333334

123456789012345678901234567890123456789012345678901234567890

LHDSPSPVRSTLGEPARSGLVDDPDMASRTPFGSASRPSAWEAPYGNANARYVPAEHPAG

555555555555555555555555555555555555555555555555555555555556

444444444555555555566666666667777777777888888888899999999990

123456789012345678901234567890123456789012345678901234567890

GAMAASIQEGIELIG---------------------------------------------

666666666666666666666666666666666666666666666666666666666666

000000000111111111122222222223333333333444444444455555555556

123456789012345678901234567890123456789012345678901234567890

----------------------------QYFDRMEREYGLNVQAPVTQPGGAAPQGPLSA

666666666666666666666666666666666666666777777777777777777777

666666666777777777788888888889999999999000000000011111111112

123456789012345678901234567890123456789012345678901234567890

LPKELLEKIADHAGPRVRRTLTAVSKPLRNAAWASTKHLTVWDKAAFGRLQNYPALESLR

777777777777777777777777777777777777777777777777777777777777

222222222333333333344444444445555555555666666666677777777778

123456789012345678901234567890123456789012345678901234567890

FHGHLSIEDLRALPPSVRHLDLSGCTGSAVSEAGLAVLARLPLESLDLSGTRIGDREVQA

777777777777777777788888888888888888888888888888888888888888

888888888999999999900000000001111111111222222222233333333334

123456789012345678901234567890123456789012345678901234567890

LASSTSLTSLNLSGNRIGNAGAQALGRNTVLTALNVSANPIGDAGVQALADSRSLTSLEL

888888888888888888888888888888888888888888888888888888888889

444444444555555555566666666667777777777888888888899999999990

123456789012345678901234567890123456789012345678901234567890

RGIGIGEAGIAALASN-TVLRSLDISSNDLSEQSAAELARNQTLASLKANACGLTNSMAQ

999999999999999999999999999999999999999999999999999999999999

000000000111111111122222222223333333333444444444455555555556

123456789012345678901234567890123456789012345678901234567890

QLARIRSLRTLEVGSNSIGDTGVLAIARNASLRTLNLSHNPITLQGLRPLELSRTLTSLD

111111111111111111111

999999999999999999999999999999999999999000000000000000000000

666666666777777777788888888889999999999000000000011111111112

123456789012345678901234567890123456789012345678901234567890

VSGIGCGDRGALLLSKNRALTSLKLGFNGIGSAGAQGLAANRTLISLDLRGNTIDVDAAK

111111111111111111111111111111111111111111111111111111111111

000000000000000000000000000000000000000000000000000000000000

222222222333333333344444444445555555555666666666677777777778

123456789012345678901234567890123456789012345678901234567890

ALANTGCLTSLNVSDCKLDDEAASALAESLTLTSLDVSVNRLSGQAARALAGNATLTSLN

111111111111111111111111111111111111111111111111111111111111

000000000000000000011111111111111111111111111111111111111111

888888888999999999900000000001111111111222222222233333333334

123456789012345678901234567890123456789012345678901234567890

I--SHNHIGPDGAQALAES---PSLTSLDARANGIGEAGARALENNTRMQGTPQNPHFLA

11111111111111111

11111111111111111

44444444455555555

12345678901234567

ENVPE------------
