# Supplementary material for: Origin and Evolution of GALA-LRR, a New Member of the CC-LRR Subfamily: From Plants to Bacteria?
Source: PLoS One. 2008 Feb 27;3(2):e1694. doi: 10.1371/journal.pone.0001694 (PMC2244805; doi:10.1371/journal.pone.0001694)
Supplement: Figure S3 — Positions of positive selection in GALA2 (0.02 MB DOC) [file pone.0001694.s004.doc]

**Figure S3, Supplemental Data, Kajava et al. 2008**

>UW551GALA2

MVAPVSTHHAPLPS**A**PAATDTGARHG

PD**Q**TGQQPAYAPARSSAGVALSPLGGLASLRLDAASANT**V**CAP

RILPPAPC**H**DPRTAALQRVTHL**S**VHDRRALGELHHYPNL**T**SLQL

EGNFTL**Q**DLKALPAT-LRHLDLSA

CTGGAKSFEAIAYLAGLPLESLNV

AGADIGDDGARLLAANPSLRALNA

ANGGIGAAGARALAESPVLASLDL

TRNGIGDEGARALADSRSLT**N**LAV

LNCLVTDVGARALA**G**N**G**TLTALDL *GNLITETGNELEQAGYDR*

TANEITARGAWALAQNRSLTSLSI

QGNLCGDGGVQALAKNRTLTSLNV

AYTDMTPASATELARNPVLTSLSV

RWNYGLGDAGVVELAKSPSLTLLDA

RSTGMGERATLALSANARIRVLHDSP**S**PVRSTLGEPARSGLVDDPD**M**ASRTPFGSASRPSAWEAPYGNANARY**V**PAE**H**PAGGAMAASIQEGI**E**LIG**Q**YFDRME**R**EYGL**N**VQAPVTQPGGAAPQGPLSA

**LPKELLEKIADHAGPRVRRTLTAVSKPLRNAAWASTKHLTVWDKAAFGRLQNYPALESL**

RFHGHLSIEDLRALPPSVRHLDL

SGCTGSAVSEAGLA**V**LARLPLESLDL

SGTRIGDREVQALASSTSLTSLNL

SGNRIGNAGA**Q**ALGRNTVLTALNV

SANPIGDAGVQALADSRSLTSLEL

RGIGIGEAGIAALASNTVLRSLDI

SSNDLSEQSAAELA**R**NQTLASLKA

NACGLTNSMAQQLARIRSLRTLEV

GSNSIGDTGVLAIARNASLRTLNL

SHNPITLQGLRPLELSRTLTSLDV

SGIGCGDRGALLLS**K**NRALTSLKL

GFNGIGSAGAQGLAANRTLISLDL

RGNTIDVDAAKALANTGCLTSLNV

SDCKLDDEAASALAESLTLTSLDV

S**V**NRLSGQAARALAGNATLTSLNI

SHNHIGPDGAQALAESPSLT**S**LDA

RANGIGEAGARALENNTRMQGTPQNPHFLAENVPE

tttttthhhhhhhhhhttttbbbb

1 11 15 21

=================================

Positions of positive selection are in red.

Region in bold is F-box domain.
